# Periodontal tissues are targets for Sars-Cov-2: a post-mortem study

**DOI:** 10.1080/20002297.2020.1848135

**Published:** 2020-11-26

**Authors:** Bruno Fernandes Matuck, Marisa Dolhnikoff, Gilvan V. A. Maia, Daniel Isaac Sendyk, Amanda Zarpellon, Sara Costa Gomes, Amaro Nunes Duarte-Neto, João Renato Rebello Pinho, Michele Soares Gomes-Gouvêa, Suzana C.O. M. Sousa, Thais Mauad, Paulo Hilário do Nascimento Saldiva, Paulo H. Braz-Silva, Luiz Fernando Ferraz da Silva

**Affiliations:** a Department of Pathology, School of Medicine, University of Sao Paulo, Sao Paulo, Brazil; b Department of Otorhinolaryngology, School of Medicine, University of Sao Paulo, Sao Paulo, Brazil; c Department of Stomatology, Division of Periodontology, School of Dentistry, University of Sao Paulo, Sao Paulo, Brazil; d Department of Stomatology, Division of Oral Pathology, School of Dentistry, University of Sao Paulo, Sao Paulo, Brazil; e Institute of Tropical Medicine of Sao Paulo, Department of Gastroenterology, LIM-07, University of Sao Paulo, Sao Paulo, Brazil; f Department of Stomatology, Division of General Pathology, School of Dentistry, University of Sao Paulo, Sao Paulo, Brazil; g Institue of Tropical Medicine of São Paulo, University of Sao Paulo, Sao Paulo, Brazil

**Keywords:** COVID-19, autopsy, infection control, oral manifestation, RT-PCR

## Abstract

**Background:** The ability of coronavirus SARS-CoV-2 to spread is one of the determinants of the COVID-19 pandemic status. Until June 2020, global COVID-19 cases surpassed 10 million. Asymptomatic patients, with no respiratory impairment, are believed to be responsible for more than 80% of the transmission. Other viruses have been consistently detected in periodontal tissues.

**Objective:** The aim of this study was to investigate the presence of SARS-CoV-2 in periodontal tissue.

**Methods:** We conducted video-endoscope minimally invasive post-mortem biopsy in seven fatal cases of COVID-19, using a regular endoscope video system associated with a smartphone to locate periodontal tissue. We analyzed the samples using RT-PCR, to identify the SARS-CoV-2 RNA and histopathological analysis.

**Results:** The seven studied autopsies with positive laboratory tests for COVID-19 included 57.14% of female patients at the average age of 47.4 (range 8 to 74). In five cases, periodontal tissue was positive for SARS-CoV-2 (RT-PCR). Histopathologic analyses showed morphologic alterations in the keratinocytes of the junctional epithelium, a vacuolization of the cytoplasm and nucleus and nuclear pleomorphism.

**Conclusion:** We presented a biomolecular analysis obtained from minimally invasive autopsies. This is the first study to demonstrate the presence of SARS-CoV-2 in periodontal tissue in COVID-19 positive patients.

## Introduction

An epidemic started in Wuhan (Hubei Province, China) with pneumonia-like symptoms, rapidly spread across the world and it was announced by the World Health Organization (WHO) as a pandemic, now called COVID-19. Its etiological agent was identified as a new coronavirus [1, 2] responsible for a severe acute respiratory syndrome. The disease leads to a diffuse alveolar damage-causing respiratory distress, and eventually death [3]. By 30 June 2020, the number of confirmed cases adds up to around 8,860,000 with more than 465,000 deaths, affecting 216 countries (https://www.who.int/emergencies/diseases/novel-coronavirus-2019).

SARS-CoV-2 spreads much more rapidly than other respiratory infections and this may be related to a long-term incubation time and the high ability of the virus to contaminate through coughing or sternutation, during social interaction [4]. Due to the low prevalence of rhinorrhea in COVID-19 patients, it is suggested that infected droplets are not only contaminated by nasal sputum and lower respiratory fluids but also by saliva [5]. Some studies suggest that the consistent findings of SARS-CoV-2 in saliva may be used as a point-of-care technology to diagnosis and prognosis, even without understanding if the virus is capable to replicate in salivary gland tissues [6].

Saliva is a biological fluid composed by salivary gland excreta, crevicular fluid, lower respiratory secretion and exfoliated epithelial cells, in which SARS-CoV-2 can be found [7]. The presence of the virus in the oral cavity may be related to different sources. SARS-CoV-2 infects cells using the angiotensin-converting enzyme 2 (ACE2) receptor as an entrance [3]. That receptor may be found in several oral sites such as tongue (Hao X et al. 2020), salivary gland ductal epithelial cells [8] and periodontal tissue [9]. The ACE2 receptor was also expressed in gingival and periodontal ligament in human fibroblasts [10]. Additionally, it was proposed that the increased protease levels in chronic periodontitis could potentially raise the risk of an oral mucosa mediated coronavirus [11].

Viral genomes of Herpes simplex virus (HSV), Epstein–Barr (EBV) and Human Cytomegalovirus (HCMV) [12] have been detected in gingival tissues [13], subgingival plaque [14] and gingival crevicular fluid [15–17]. The possible sources of the infection could be the gingival epithelial cells exposed to the oral cavity and virus migration through the bloodstream [18]. It has been hypothesized that the periodontal pocket could be a niche for new coronavirus, due to a favorable environment to replicate and eventually migrate systemically using the capillary periodontal complex [1]. Moreover, predisposing diseases, such as diabetes mellitus, hypertension, cardiovascular diseases and metabolic syndrome, that can contribute to a worse prognosis of COVID-19 are highly associated with periodontal disease, making possible an association between periodontal disease and COVID-19 [19]

Due to the risk of contagion, tissues analyses from oral sites have been small, the presence of infected saliva and pulmonary fluid mitigated the possibility of biopsies or autopsies in these organs. In this study, we describe a minimally invasive procedure that reduces the direct contact with the oral cavity and could be a way to understand COVID-19 mechanisms in the oral cavity reducing the chance of contamination.

## Methods

This study was approved by the institutional and federal ethics board, protocol number 30364720.0.0000.0068. The minimally invasive autopsy was performed after informed consent from the next-of-kin.

Deceased patients with SARS-CoV-2 positive test (nasopharyngeal swabs) were submitted to minimally invasive autopsy – videoscope-guided. These procedures were performed at the PISA Research Center, University of Sao Paulo Autopsy Service and University of Sao Paulo School of Medicine.

### Safety protocol

We performed a minimally invasive autopsy with ultrasound-guided post-mortem biopsies to obtain samples, following established safety protocols previously described [20].

The deceased bodies were wrapped in a safety plastic bag, the access to the autopsy room was restricted to three health-care professionals using appropriate personal protective equipment. The professionals were tested for SARS-CoV-2 using PCR and all team members were negative throughout 60 days of procedures.

### Sampling protocol

**Figure 1.**
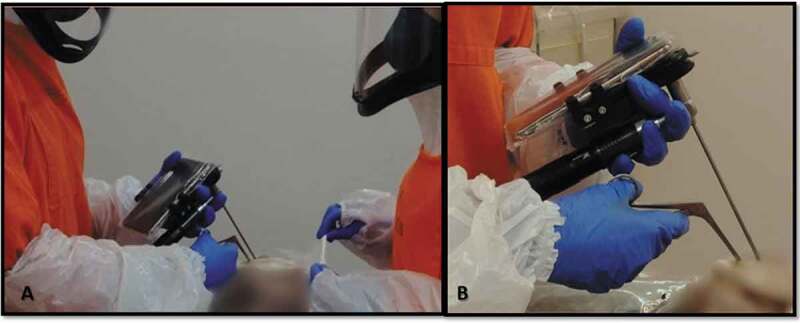
Minimally invasive autopsy – Videoscope Guided – **A**. Two healthcare persons with personal protective equipment (PPE) during autopsy procedure **B**. Videoscope (MScope, Karl-Storz Optical 4 mm, 30º) attached to a smartphone for visual inspection and sampling removal

A multidisciplinary team, constituted by an oral and maxillofacial pathologist, an otorhinolaryngologic and an autopsy technician, performed the minimally invasive autopsy. We used a regular endoscope video system (MScope – Karl-Storz Optical, Tuttlingen – Germany), associated with a smartphone, to achieve the oral cavity and localize periodontal tissues (Figure 1).

All autopsies were performed in a similar procedure: an incision of 15 cm was made in a plastic safety bag that involved the patient; this opening was performed at the upper lip region, to allow the access of endoscope to intraoral sites. The optical endoscope was used to locate periodontal tissue – the interproximal mesial papilla of the first superior molar – in two of the cases the patient had no molars and the tissue collected was from the next mesial tooth available.

Considering the absence of salivary flow, we used gauze soaked in an enzymatic detergent (Riozyme – Rioquímica, Brazil) to clean superficial contamination from the gingival biopsy area. The Molt periosteal elevator in a prying motion was used to elevate interproximal tissue and Takahashi forceps were applied to clamp and collect tissue samples from the site, using an endoscope as a visual guide.

Once periodontal tissues had been removed, two fragments were dissected in the longitudinal axis, resulting in two similar parts. One of them was formalin-fixed to histopathological analyses and the other one was frozen (−80ºC) and sent to molecular analyses.

Once tissues were collected the opening in the plastic bag was closed with a transparent adhesive to mitigate the risk of contagion.

### Histological and molecular diagnosis of SARS-CoV-2

Tissue samples were fixed in buffered 10% formalin, embedded in paraffin and 3 µm sections were stained with hematoxylin and eosin (H&E). Samples measuring 0.5 cm^3^ were stored at −80°C. Tissue samples were macerated, and nucleic acid extraction was performed using the TRIzol® reagent (Invitrogen). Molecular detection of SARS-CoV-2 was performed with the use of the SuperScriptTM III Platinum^TM^ One-Step qRT-PCR Kit (Invitrogen) and primers/probes sets for E, RdRp and N (N1) gene amplification [21,22]. Human RNase P gene was also amplified as a nucleic acid extraction control [22].

We examined specimens from periodontal tissue – including the junctional epithelium, adjacent oral gingival epithelium and connective tissue from fatal cases of COVID-19. Using rRT-PCR we investigated the presence of SARS-CoV-2 RNA in tissues and correlated it with clinical conditions of the patients, since their first symptoms, their ICU time and autopsy findings.

rRT-PCR reactions were performed using the 7500 Fast Real-Time PCR System (Applied Biosystems) and consisted of a step of reverse transcription at 55°C for 10 min for reverse transcription, 95°C for 3 min and 45 cycles at 95°C for 15 s and 58°C (E and RdRp genes)/55°C (N and RNAse P genes)/for 30 s.

## Results

We included in this study 7 (seven) patients (three men/four women). All patients tested positive for SARS-CoV-2 by nasopharyngeal swabs. The mean age was 47.43 y (8–74 y), and the average number of days between the first symptoms and death was 20.14 days (10–31 days).

All patients presented with the severe acute respiratory syndrome and were admitted in the Hospital das Clinicas of Medicine School of Sao Paulo University ICU for mechanical ventilation support. The most frequent symptoms were fever, cough and dyspnea.

Most of the patients presented at least one preexisting comorbidity like diabetes mellitus, systemic arterial hypertension, malignant neoplasm, cardiovascular disease, asthma or any immunosuppressive condition. There was only one patient free of any comorbidities. Only non-smoker patients were included in this study (Table 1).

**Table 1.** Characteristics of patients included in this study and results of SARS- CoV-2 detection in periodontal tissues using an E-gene primer/probe set

Case number	Gender	Age	DM	SAH	Smoker	Period of hospitalization (days)		Time from symptoms onset to death (days)	CT PCR-RT Periodontal tissue	Nuclear pleomorphism	Vacuolization
1	F	51	No	No	No		14	31	Negative	No	No
2	F	71	Yes	Yes	No		11	16	29.41	No	Yes
3	F	15	No	No	No		9	24	33.23	No	No
4	F	74	No	Yes	No(Ex-smoker)		9	23	27.28	Yes	No
5	M	64	Yes	Yes	No		12	14	30.47	No	No
6	M	49	Yes	No	No		13	23	36.55	Yes	Yes
7	M	8	No	No	No		6	10	Negative	No	Yes

DM: Diabetes mellitus.

SAH: Systemic arterial hypertension.

CT-PCR-RT: Cycle threshold – Polymerase Chain Reaction – Real time.

**Figure 2.**
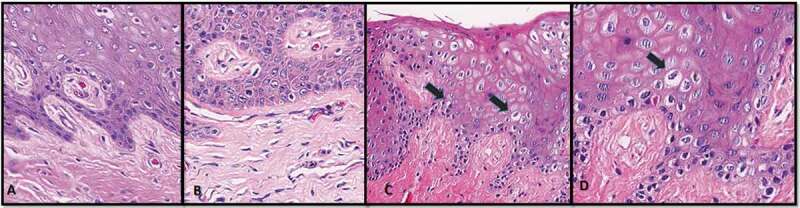
Histopathological assessment – **A**. Junctional epithelium, no infiltration by inflammatory cell is present (20 x) **B**. Cellular and nuclear pleomorphism (20 x) **C**. Cellular vacuolization (20 x – arrow) D. Junctional epithelium with intracellular edema and parabasal vacuolization (40 x – arrow)

Histopathologic analyses showed morphologic alterations in the keratinocytes of the junctional epithelium, characterized mainly by vacuolization of the cytoplasm and nucleus, and sometimes nuclear pleomorphism (Figure 2). Lung samples presented exudative and proliferative diffuse alveolar damage (DAD), with epithelial atypia which extended throughout the respiratory epithelium as described by 20.

We detected SARS-CoV-2 by rRT-PCR in 5/7 samples of periodontal tissues with a mean cycle threshold (Ct) value (E primer/probe sets) of 31.38 (27.28–36.55).

## Discussion

We present the molecular and histopathological features observed in seven autopsies of COVID-19 patients in Brazil, five periodontal tissues from deceased patients were positive. Of the two negative patients, one was an 8 years old boy. The pathological aspects of COVID-19 in children are still unclear, the disease seems to take a mild course. One of the explanations is related to the differences in ACE2 receptor expression in children [23], that can corroborate with the absence of viral genome in periodontal tissue. The presence of vacuolization may be related to complication during the hospitalization time. The other patient had thewoman with the longest time between the first symptoms and death, the long-term hospitalization time could make possible a clarification of the virus present in the periodontal cells of the host.

Autopsy is an important tool to understand the pathological mechanisms of viral diseases. We have had a recent experience in Brazil from other two outbreaks of viral infections (yellow fever and zika) and thus it demanded our research group to develop new autopsy procedures to study and contribute with physicians and decision makers [23]. The ultrasound-guided minimally invasive autopsy is a reliable alternative to conduct autopsies on COVID-19 cases because it considerably reduces the costs and the production of aerosols [23]. This is the first study to associate oral autopsy findings with a minimally invasive procedure – videoscope guided – as a new approach to study viral/pandemic, highly contagious diseases and its oral manifestations.

Saliva and gingival crevicular fluid have been shown to be sources for human viruses in the oral cavity. A recent study investigating the detection of human herpesviruses in saliva and gingival crevicular fluid in patients with chronic kidney disease found different prevalences between these two sites [17]. The same was founded during Zika virus international public health emergency; peptides were identified in saliva of showing a new transmission path of the disease [24]. Although our research did not analyze the components of saliva or crevicular fluid, we observed the presence of SARS-CoV-2 RNA copies in the periodontal tissue even many days after the first symptoms. This finding may justify the oral cavity as a source of SARS-CoV-2, as it has been consistently detected in saliva [7, 25, 26, 27], suggesting that it may be related to the access via cavity-specific crevicular fluid.

In the presented cases, the viral infection seems to be in a longstanding pattern, suggesting that even in patients who had a long course of the disease, viral infection in periodontal tissue persists. The presence of a high viral load of SARS-CoV-2 in saliva shortly after symptoms of COVID-19 is already known [28, 27]. Similar to what we found, To et al. have also reported that contagious particles of viral RNA could still be detected in saliva samples from some patients for 20 days or longer after the first symptoms. Our data showed viral RNA of SARS-CoV-2 in periodontal tissue until 24 days after the first symptoms in some patients.

Other studies trying to indicate potential targets for SARS-CoV-2 showed that cells from brain, coronoid plexus [29] and kidney can be a source [30,31,32,33] suggesting that organotropism can indicate the course that the disease is going to take. In this study, we evaluated a possible organotropism that can influence transmissibility. Saliva is the major component of droplets and responsible for the high contagion pattern of COVID-19. In this context, the presence of SARS-CoV-2 on periodontal tissue can be one of the components that contributes with the saliva viral load.

Detection of SARS-CoV-2 RNA in the periodontal tissues draws our attention to possible implications of periodontal treatment for patients with COVID-19. Supra- and subgingival debridement, even without aerosol generation, can be potentially contaminating events. Ultrasonic scalers, air-water syringes and hand-pieces for maintenance therapy and root planning are more likely to facilitate transmission due to the spray that can contain particles in droplets of saliva, gingival crevicular fluid, blood and other debris. The Centers for Disease Control and Prevention (CDC, USA) (https://www.cdc.gov/coronavirus/2019-ncov/hcp/dental-settings.html) recommend that dental settings should prioritize urgent visits and follow following strict biosafety protocols. Clinicians and dental staff should be aware of the importance of using personal protective equipment, disinfection and sterilization.

The results of this investigation should be interpreted with caution due to some limitations. First, our observations are based only on seven cases. Possibly, a greater understanding of the presence of the virus in the gingival crevicular fluid and periodontal tissues will emerge as new findings when larger numbers of cases are reported. Furthermore, the fatal cases described here represent severely ill patients with COVID-19 with prolonged periods of hospitalization, mechanical ventilation and enteral feeding in critical care, requiring oral and nasal tubes, which could explain the histopathological changes observed. Finally, it is possible that the periodontal tissue response is different in individuals with COVID-19 who are asymptomatic or have only mild symptoms.

We conclude that oral minimally invasive autopsy – videoscope guided – is a safe procedure to obtain samples that can be analyzed by biomolecular and histopathological assays, including autopsies realized during a highly contagious pandemic situation. This is the first study to perform oral minimally invasive autopsies and our findings show that periodontal tissue seems to be a target for SARS-CoV-2, and can contribute for a long time to the presence of the virus in saliva samples. These findings can indicate a new approach to understand the contamination pattern of COVID19.
